# Topography and life stage regulate species aboveground biomass distribution in combination in a tropical montane rainforest

**DOI:** 10.3389/fpls.2024.1403048

**Published:** 2024-09-02

**Authors:** Jiaming Wang, Han Xu, Yide Li, Yanpeng Li

**Affiliations:** ^1^ Hainan Jianfengling National Key Field Research Station for Tropical Forest Ecosystem, Research Institute of Tropical Forestry, Chinese Academy of Forestry, Guangzhou, China; ^2^ School of Water Resources and Environment Engineering, Nanyang Normal University, Nanyang, China; ^3^ School of Ecological and Environmental Sciences, East China Normal University, Shanghai, China

**Keywords:** topography, species aboveground biomass, life stages, topographic preferences, species-topography associations change

## Abstract

Understanding the correlation between topography, species biomass and species life stage would allow forest managers to better foster carbon storage in forests. Using census data from a 60-ha plot in south China, we first quantified aboveground biomass (AGB) and how much it varied among different topographies. Next, the specific contribution of 42 dominant species to total aboveground biomass was analyzed for each of the different topographies. We also explored whether these species-topography associations changed, in terms of species’ AGB distribution, during each of the three life stages (sapling, juvenile, adult) for these 42 species. Our results showed that the average AGB was 368.79 Mg ha^-1^ and that it varied noticeably among the four topographies (Low valley, Slope, High valley and Ridge, which were classified by using fuzzy C-mean clustering based on elevation, convexity, and slope). AGB was significantly lower in the two valleys than in the two other topographies. Of the 42 species, 88.1% showed topographic preferences, and 78.6% appeared to exhibit topographic preferences that changed with life stage. Our work highlights the importance of topography and life stage in species biomass distribution and suggests that different combinations of species and life stages, based on species topographic preferences across life stages, may be better suited in different tropical rainforest topographies to maximize carbon storage overall.

## Introduction

Globally, forests are a major reservoir of carbon, and they can act either as a carbon source when logged or disturbed or as a carbon sink when recovering post-disturbance. When forests accumulate carbon, they mitigate the global carbon emissions that cause an imbalance in the atmosphere by accumulating biomass through photosynthesis (Deal et al., 2012; Zhao et al., 2016). Therefore, forests play important roles in the regional and global carbon cycles as well as in the corresponding maintenance of biomass (Lorenz and Lal, 2010; Pan et al., 2011). Still, forests are sensitive to disturbance and varies greatly throughout heterogeneous habitats (Lorenz and Lal, 2010; Lin et al., 2012; Ratcliffe et al., 2017).

As key environmental factors, topographical factors such as elevation, convexity and slope strongly influence local-scale variation in microclimate, hydrological conditions and soil nutrient availability (Werner and Homeier, 2015; Jucker et al., 2018; Wang et al., 2023). As such, they constrain the conditions within which plant grows, driving environmental filtering, controlling the distribution of species, and influencing the biomass of forests (Mascaro et al., 2011; Riihimäki et al., 2017; Jin et al., 2018; van der Plas, 2019; Yuan et al., 2020). Many studies have demonstrated significant associations between trees and topography in forests (Gunatilleke et al., 2006; Fortunel et al., 2018; O'Brien and Escudero, 2022; Zuleta et al., 2020, 2022). Some tree species have been found to be more or less frequent in swamp, valley, slope, ridge, or low-lying plateau habitats (Harms et al., 2001; Queenborough et al., 2007; Guo et al., 2015). However, species abundance does not necessarily relate to biomass. Information regarding how tree species-specific contributions to forest aboveground biomass vary among different topographies is scarce, though this knowledge would allow for a novel way to assess species coexistence and would provide valuable information to forest managers looking to maximize carbon storage (Lin et al, 2012; Colgan and Asner, 2014; Smith et al., 2016).

The concept of the regeneration niche suggests that species-topography associations change with life stage because each stage has different physiological requirements and responds to different selective pressures (Grubb, 1977; Webb and Peart, 2000; Silvertown, 2004; Cui and Zheng, 2016; Yao et al., 2020). Species-topography associations change can be quantified by counting the individuals of a species at different life stages, as measured by tree DBH (diameter at the breast height) size (Comita et al., 2007; Lai et al., 2009; Kanagaral et al., 2011). Also important is detecting how these associations change based on species’ aboveground biomass during different life stages. Species aboveground biomass encompasses species abundance and tree volume with wood density, but it also expresses the outcome of myriad ecological processes (De Castilho et al., 2006; Colgan and Asner, 2014). This knowledge could further be beneficial for understanding species coexistence (Colgan and Asner, 2014; Ramachandran et al., 2023) and for promoting biomass carbon gain in controlled burns.

Tropical rainforests, with their high levels of biodiversity, are among the most productive ecosystems on the earth (Field et al., 1998). Here, we utilized the 60-ha forest dynamic plot that was established in the primary tropical montane rainforest in Hainan, China to analyze the correlations between topography, species aboveground biomass (AGB) and life stage. Our objectives were 1) to quantify AGB and its variation among topographies; 2) to determine the species-topography associations based on species-specific AGB contribution; and 3) to analyze how species-topography associations change across life stages based on species AGB distribution.

## Materials and methods

### Study site

This study was conducted in the 60-ha (1 000 m × 600 m) Forest Dynamics Plot (FDP) established between 2009 and 2010 in Jianfengling National Nature Reserve (JNNR: 18.33°~18.95°N, 108.68°~109.20°E), Ledong County, Hainan Province, China. The site is characterized by a tropical monsoon climate, with an average annual precipitation of 2449 mm, and a mean annual temperature of 19.8° (Zhou et al., 2009). The main soil type is lateritic yellow soil with high soil moisture and humus content (Li et al., 2002).

### Data collection

The Jianfengling FDP was divided into 1500 20 m × 20 m quadrats using 2464 posts. Plot altitude varies from 866.3 m to 1016.7 m, convexity varies from -7.7 to 7.2, and slope ranges from 1.7° to 49.3°. Between December 2010 and December 2011, all woody plants with DBH ≥ 1.0 cm were mapped, tagged, measured, and identified to species. Overall, a total number of 439,676 stems from 62 families, 155 genera, and 290 species were recorded (Xu et al., 2015a). The four most dominant families were Lauraceae, Fagaceae, Rubiaceae and Arecaceae, and the importance values for these families were all greater than five. The four most dominant species were *Livistona saribus*, *Gironniera subaequalis*, *Cryptocarya chinensis*, and *Alseodaphne hainanensis*.

### Topography classification

**Figure 1:**
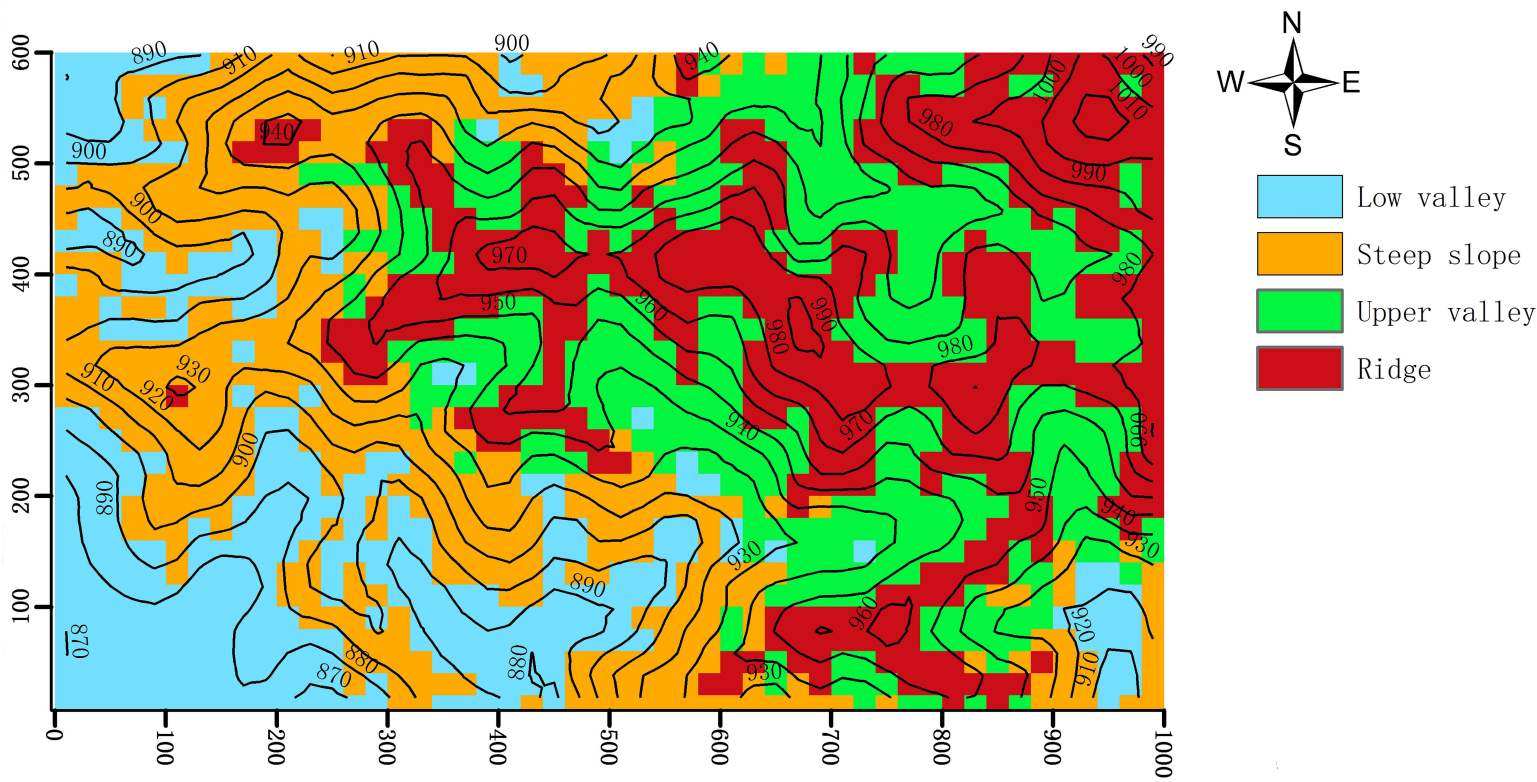
Map of the distribution of the four topographies of the Jianfengling 60 ha plot, as classified by fuzzy C-mean clustering in 20 m×20 m cells.

We classified the Jianfengling FDP into four topographies (**Figure 1**; Low valley, Steep slope, Upper valley, Ridge) by using fuzzy C-mean clustering based on elevation, convexity, and slope in each of the 20 m×20 m quadrats (Wang et al., 2018; **Table 1**). Fuzzy C-means clustering obtains the membership degree of each sample to all cluster centers by optimizing the objective function, and then can automatically classify sample data. Among fuzzy algorithms, it is the most widely used and successful because it can reflect the real world objectively (Gong et al., 2007; Bezdek, 2013).

**Table 1:** The characteristic and quadrats number of 4 topographies.

Topography	Elevation /m	Slope /(°)	Convexity /m	Mean elevation /m	Mean slope /(°)	Mean convexity /(m)	Quadrats’ number
Low valley	894.47	19.41	-1.19	892.26 ± 15.11	18.24 ± 6.94	-1.35 ± 1.94	315
Steep slope	918.44	27.75	0.59	916.45 ± 13.63	28.51 ± 5.80	0.93 ± 1.73	417
Upper valley	948.56	25.54	-1.17	949.75 ± 14.71	25.13 ± 7.35	-1.88 ± 1.50	377
Ridge	968.30	25.70	2.08	970.03 ± 17.39	25.46 ± 7.26	2.37 ± 1.58	391

The values of elevation, slope, and convexity in the 2 to 4 columns were the value of cluster centers clustered by fuzzy C-mean clustering. Mean value= Mean value ± SD (a standardized deviation).

### Aboveground biomass calculation

In this study, the AGB of each tree was estimated using the allometric model for the tropical montane rainforest in Jianfengling (Equation 1) (Zeng et al., 1997; Wang, 2017). With this equation, we calculated AGB for each tree in the Jianfengling FDP.


(1) $$\text{AGB }=0.100194058\times {\text{DBH}}^{2.463324542}{\text{, R}}^{2}=0.94$$

Zeng et al. (1997) fitted an allometric growth equation of more than 70 tree species in Jianfengling. On this basis, Equation 1 was fitted by adding more than two hundred analytic trees. This mixed-species allometric regression model has a high accuracy due to the large number of species considered and the large number of samples, which offset the inter-species differences. In addition, in tropical forests, the dominant species are not obvious. If the regression model of each tree species is fitted, the workload is huge. Given its convenience and high accuracy, we used Equation 1 to calculate AGB.

### Life stage classification

D95_0.1_ represents the 95th percentile of DBH of all individuals in a species≥0.1D_max_, where D_max_ is the largest measured DBH in a species (King et al., 2006). Previous studies have verified that D95_0.1_ can be widely applied to the division of the adult stage of trees in old-growth forests (King et al., 2006; Lan et al., 2011). Therefore, individuals of each of 42 canopy species were divided into three life stages according to D95_0.1_: saplings [(1, 5) cm DBH], juveniles [(5, D95_0.1_) cm DBH], and adults (>D95_0.1_ cm DBH).

### Relative contribution of dominant species to AGB in different topographies

Our analysis focused on the dominant species ranking of the top 30 species in aboveground biomass in each of the four topographies. A total of 42 canopy species were included in this analysis because some species were present in multiple topographies. For each of the 42 species, the relative contribution to total AGB was calculated at the whole plot and at the topography scale. We examined the associations between topography and the AGB distribution of these 42 species (i.e. for each of these 42 species, whether a given species contributed significantly more or less to one or more topographies) using torus-translation tests (Harms et al., 2001; Lin et al., 2012), which consider the spatial autocorrelation in both the topography and the plant distributions. This method generates a null model in which the species are distributed randomly with respect to topography, and then it tests whether the observed relative contribution of a focal species in each topography is significantly higher or lower than would be expected at random. Further details of this method can be found in Harms et al. (2001).

Torus-translation tests were also conducted for the 42 species to examine species-topography associations across different life stages (Lai et al., 2009; Oktavia and Jin, 2019). To better understand how species-topography associations changed during three life stages (sapling, juvenile, adult), we analyzed it in two aspects. On the one hand, we explored how many species showed inconsistent associations with a specific topography across three life stages and how many showed consistent associations. On the other hand, we compared the differences in the number of significant associations with each topography among the three life stages.

In addition, one-way analysis of variance model (ANOVA) and multiple comparisons were used to test differences in AGB in different topographies. To further test the effect of topography, life stages and species on AGB distribution, two-way and three-way ANOVA were also used. All the analyses in this study were conducted in R version 2.15.1 (Oksanen, 2011).

## Results

### Aboveground biomass and its variation

**Figure 2:**
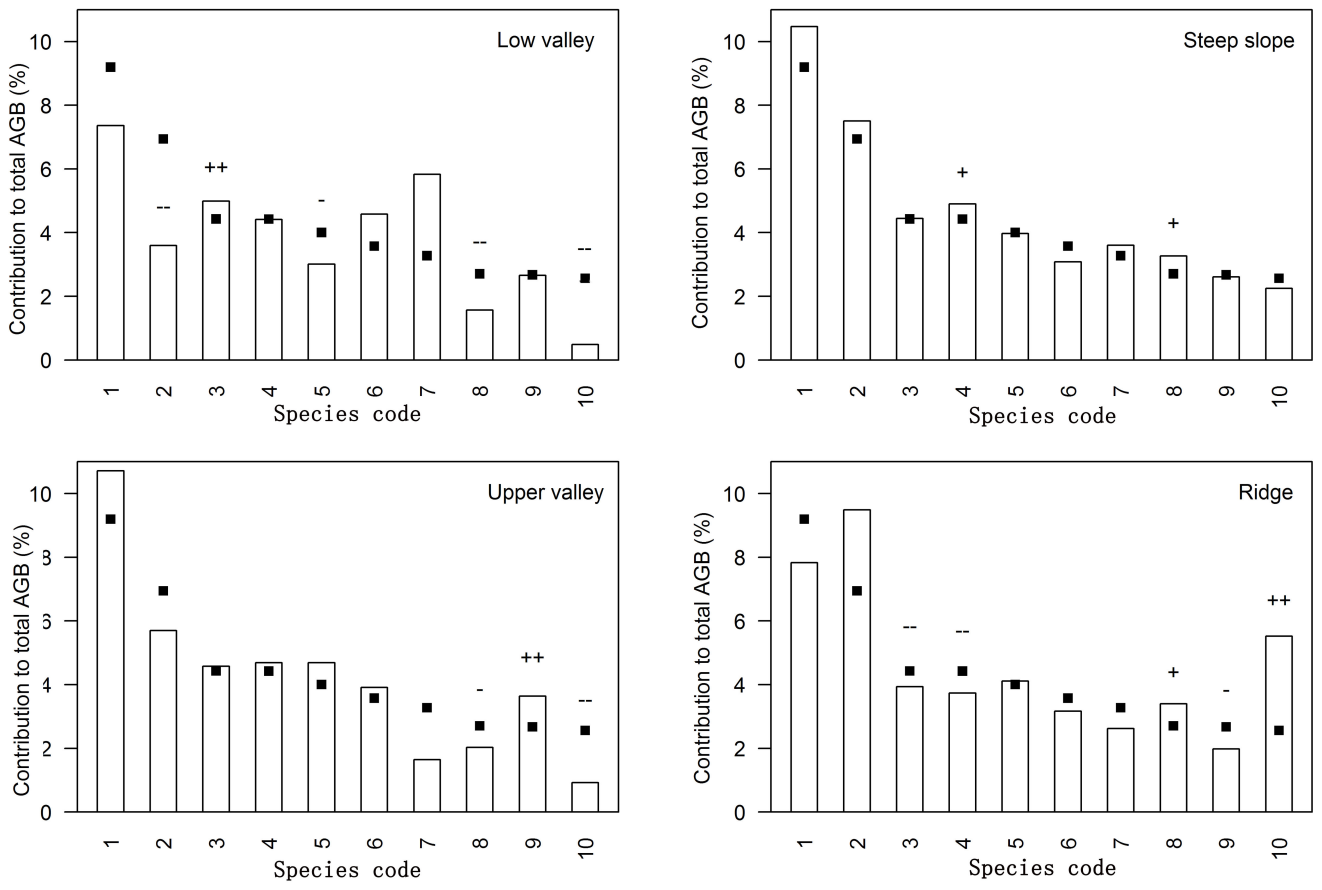
Relative contribution to total aboveground biomass (AGB) per topography type for the ten most important species contributing to the total AGB in Jianfengling FDP. Species codes are defined in **Table 3**. Bars show the relative contribution of the species to the aboveground biomass in each topography type, while squares indicate the relative contribution of the species to the aboveground biomass of the entire plot; thus differences between bars and squares represent the influence of topography associations. ‘+’ and ‘++’ respectively indicate significant positive associations at levels of 0.05 and 0.01, and ‘-’ and ‘–’ respectively indicate significant negative associations at levels of 0.05 and 0.01.

In the Jianfengling FDP, the average basal area and average aboveground biomass were 56.04 m^2^·ha^-1^ and 368.79 Mg·ha^-1^, respectively (**Table 2**). The top three species in AGB across the entire plot were *Livistona saribus*, *Lithocarpus fenzelianus*, and *Gironniera subaequalis* (**Table 3**; **Figure 2**). Just 42 of 290 species contributed to 80.3% of the total aboveground biomass in the Jianfengling FDP (**Table 3**). Average basal area and average aboveground biomass varied among the four topographies (**Table 2**). Steep slope and ridge had higher aboveground biomass than the low valley and upper valley did (P<0.05). In terms of basal area, the order was ridge > steep slope > high valley > low valley (P<0.05).

**Table 2:** Basal area and aboveground biomass in each of the four topographies and the entire plot.

Topographies	Area (ha)	Basal area (m^2^ ha^-1^)	AGB (Mg ha^-1^)
Low valley	12.60	48.15 ± 0.80 e	322.25 ± 7.00 c
Steep slope	16.68	59.96 ± 0.79 b	393.42 ± 6.86 a
Upper valley	15.08	50.70 ± 0.83 d	339.31 ± 7.38 c
Ridge	15.64	63.38 ± 0.75 a	408.48 ± 6.67 a
Entire plot	60.00	56.04 ± 0.43 c	368.79 ± 3.62 b

AGB, aboveground biomass. The values in the third and fourth columns are the means ± one standard error of means. The different lowercase letters indicate significant differences among the four topographies.

**Table 3:** Maximum DBH, AGB and tree number of 42 dominant species in Jianfengling FDP.

Code	Species	Maximum DBH (cm)	AGB (Mg)	Percentage to total AGB (%)	Number of trees
1	*Livistona saribus*	99	2034.62	9.20	3334
2	*Lithocarpus fenzelianus*	145	1536.14	6.94	1751
3	*Gironniera subaequalis*	53.7	979.93	4.43	10035
4	*Alseodaphne hainanensis*	113	979.10	4.42	5301
5	*Lithocarpus amygdalifolius*	133.8	885.85	4.00	1360
6	*Cyclobalanopsis patelliformis*	132.9	791.05	3.58	1207
7	*Altingia obovata*	120	724.78	3.28	1793
8	*Schima superba*	96	598.72	2.71	2529
9	*Alstonia rostrata*	95	591.54	2.67	1952
10	*Madhuca hainanensis*	109.2	567.45	2.56	1534
11	*Parakmeria lotungensis*	122.3	542.91	2.45	584
12	*Xanthophyllum hainanense*	68.2	490.02	2.21	6641
13	*Cryptocarya chinensis*	54.2	467.34	2.11	16811
14	*Nephelium topengii*	64.8	442.74	2.00	11873
15	*Beilschmiedia tungfangensis*	65.6	393.54	1.78	10644
16	*Michelia mediocris*	115.3	385.89	1.74	1021
17	*Cinnamomum rigidissimum*	135	382.89	1.73	5191
18	*Castanopsis carlesii*	96.8	358.44	1.62	3269
19	*Canarium album*	79.6	337.36	1.52	2533
20	*Dacrydium pectinatum*	111.8	316.73	1.43	250
21	*Daphniphyllum paxianum*	150	301.42	1.36	208
22	*Ilex goshiensis*	55.8	272.06	1.23	4548
23	*Lithocarpus pseudovestitus*	104.5	250.53	1.13	2427
24	*Castanopsis fissa*	104.1	221.03	1.00	2803
25	*Platea parvifolia*	77.8	213.70	0.97	6419
26	*Manglietia fordiana*	98.7	210.59	0.95	948
27	*Neolitsea ellipsoidea*	88.8	197.66	0.89	15739
28	*Castanopsis jianfenglingensis*	70.4	179.79	0.81	2297
29	*Dacrycarpus imbricatus*	94.4	179.78	0.81	285
30	*Endospermum chinense*	126	172.54	0.78	267
31	*Pouteria annamensis*	115	170.93	0.77	586
32	*Artocarpus styracifolius*	53.5	163.99	0.74	1243
33	*Syzygium championii*	83.5	162.70	0.74	1555
34	*Reevesia thyrsoidea*	83.8	159.60	0.72	2377
35	*Schefflera heptaphylla*	64.7	158.31	0.72	1145
36	*Machilus cicatricosa*	101.7	154.75	0.70	1651
37	*Castanopsis tonkinensis*	80.2	149.55	0.68	953
38	*Cyclobalanopsis fleuryi*	95	147.44	0.67	568
39	*Polyosma cambodiana*	41.4	145.84	0.66	4471
40	*Beilschmiedia laevis*	54.9	138.28	0.62	2602
41	*Sarcosperma laurinum*	67.2	122.75	0.55	1128
42	*Lithocarpus longipedicellatus*	80	90.90	0.41	2842

### Topographic differences in species-specific contributions

42 dominant species contributed 77.5%-87.3% of total aboveground biomass in each topography (**Supplementary Table S1**; **Figure 2**). Relative contributions of species varied among different topographies (**Supplementary Tables S1**, **S2**; **Figure 2**). 37 of the 42 species were significantly associated with one or more topographies (**Supplementary Table S2**; **Figure 2**). There were respectively 11, 4, 10, and 10 species positively associated with low valley, steep slope, upper valley, and ridge. Four species were positively associated with low valley and upper valley, and one species was positively associated with steep slope and ridge. In other words, 11, 4, 10, and 10 species contributed significantly more to low valley, steep slope, upper valley and ridge, respectively, than would be expected at random. Four species contributed significantly more to low valley and upper valley, and one species contributed significantly more to steep slope and ridge than would be expected at random. In addition, 14, 7, 7, and 14 species contributed significantly less to low valley, steep slope, upper valley and ridge, respectively, than would be expected at random. Four species contributed significantly less to low valley and upper valley, and four species contributed significantly less to steep slope and ridge than would be expected at random. The results of ANOVA indicated species had the largest effect on AGB distribution (**Table 4**). The effects of topography and their interaction effect were also significant.

**Table 4:** Variation analysis of the effects of topography and species on AGB distribution.

	Df	Sum Square	Mean Square	F value	P value
Topography	3	6.27E+07	20902907	27.663	<2e-16 ***
Species	41	4.25E+09	103614121	137.124	<2e-16 ***
Topography × Species	123	7.96E+08	6471786	8.565	<2e-16 ***

Df, degree of freedom.

*** represents significant effects at a level of 0.001.

### Variability of species-topography associations across life stages

**Figure 3:**
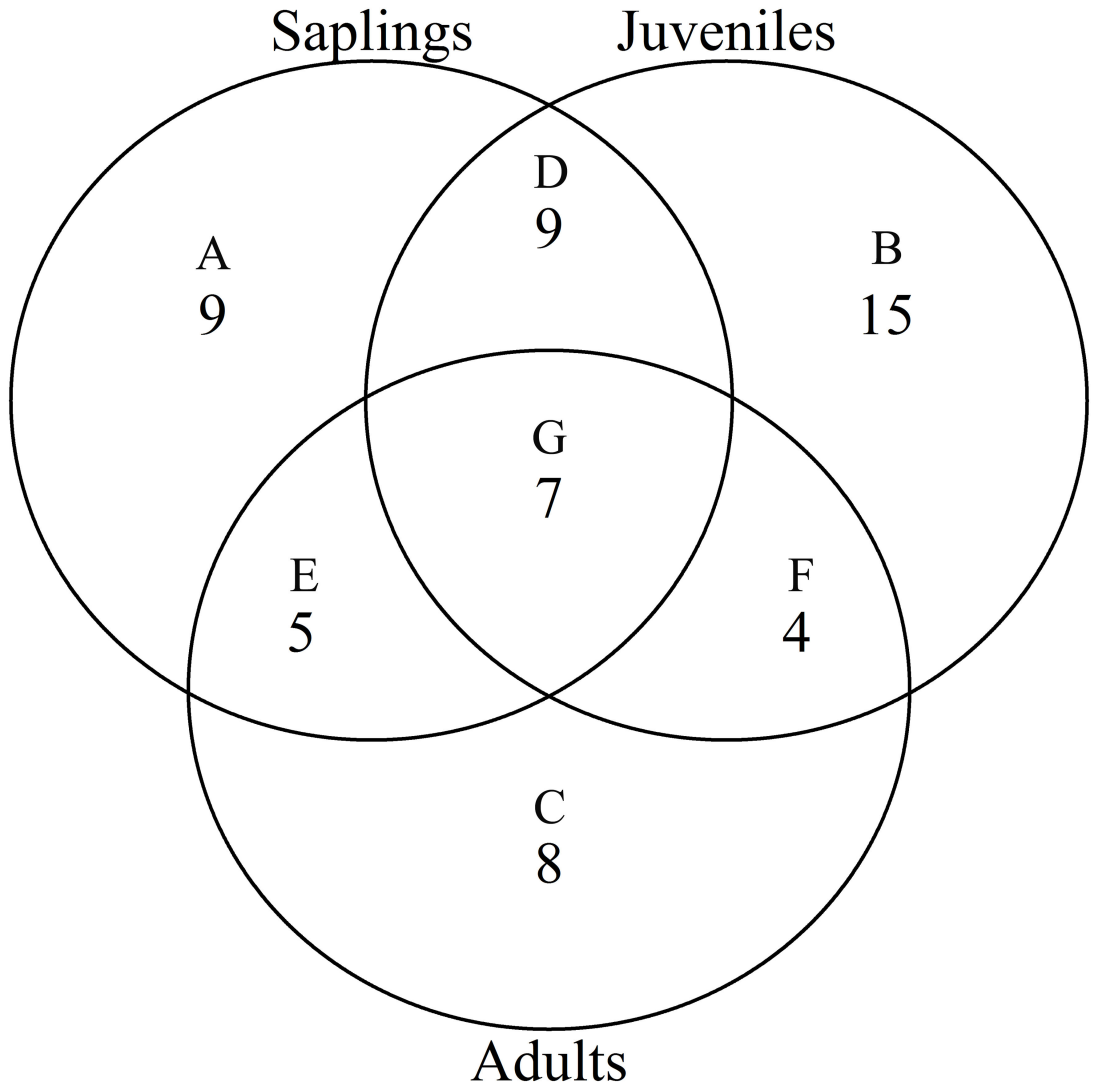
Venn diagram of positive associations of species at each life stage with all four topographies in the Jianfengling FDP. Here shown is the number of positive associations instead of the number of species. **(A–C)** show number of only one positive association at any life stage (P<0.05). **(D–F)** represent number of consistent positive associations at two stages (P<0.05), and **(G)** is number of consistent positive associations across all three life stages (P<0.05).

**Figure 4:**
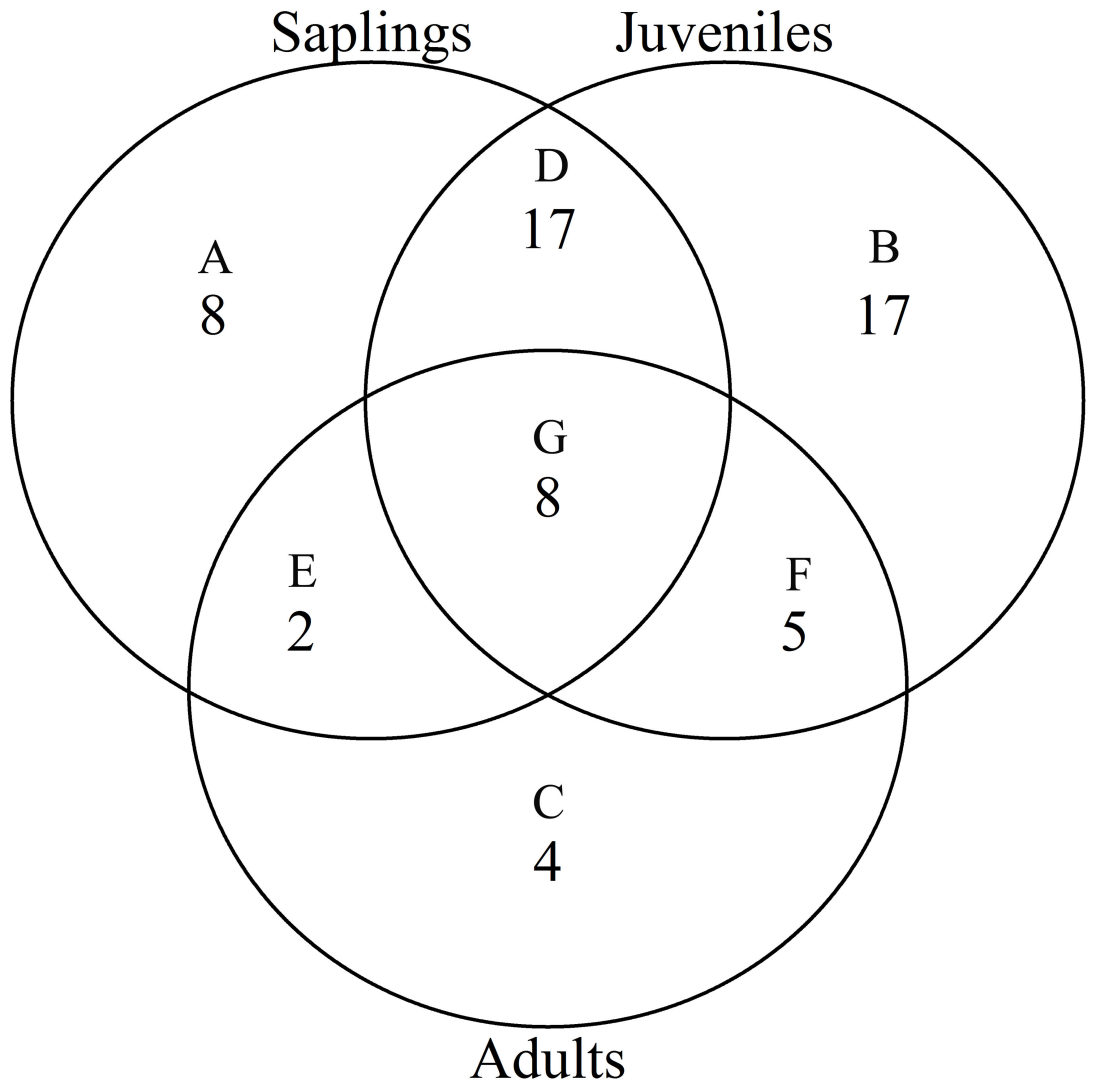
Venn diagrams of negative associations of species at each life stage with all four topographies in the Jianfengling FDP. Here shown is the number of negative associations instead of the number of species. **(A–C)** show number of only one negative association at any life stage (P<0.05). **(D–F)** represent number of consistent negative associations at two stages (P<0.05), and **(G)** is number of consistent negative associations across all three life stages (P<0.05).

According to the torus translation tests based on species AGB distribution, 89 positive associations among 38 species and 101 negative associations among 39 species were observed across the three life stages (**Figures 3**, **4**). In terms of positive associations, 16 out of 30 significant associations (D and G in **Figure 3**) between saplings and topography persisted into the juvenile stage in the same topography, and 12 of the associations (E and G in **Figure 3**) were sustained in the adult stage in the same topography. Correspondingly, 25 out of 35 negative associations between saplings and topography (**Figure 4**) persisted into the juvenile stage, and 10 of the associations were sustained in the adult stage. In summary, less than one-third of the positive associations and just over a quarter of the negative associations between saplings and topography coincided with the two later life stages (F and G in **Figure 3**). Of the adults, for one-third of positive associations and for less than one-fifth of negative associations, there were no corresponding associations between species and topography with the two prior life stages. Just 7 positive and 8 negative associations between species and topography (G in **Figures 3**, **4**) were consistent across the three life stages. In total, 33 species showed inconsistent associations with a specific topography across three life stages, and 8 species showed consistent associations. In addition, the results of ANOVA indicated life stages had the largest effect on AGB distribution, followed by species (**Tables 5**, **6**). The effects of topography and its interaction effect with species and life stages were also significant.

**Table 5:** Variation analysis of the effects of life stages and species on AGB distribution.

	Df	Sum Square	Mean Square	F value	P value
Life stages	2	1.45E+09	724896729	2878.47	<2e-16 ***
Species	41	1.42E+09	34506456	137.02	<2e-16 ***
Life stages × Species	82	1.69E+09	20603944	81.82	<2e-16 ***

Df, degree of freedom.

*** represents significant effects at a level of 0.001.

**Table 6:** Variation analysis of the effects of topography, life stages and species on AGB distribution.

	Df	Sum Square	Mean Square	F value	P value
Topography	3	2.08E+07	6933004	27.868	<2e-16 ***
Life stages	2	1.45E+09	7.25E+08	2913.847	<2e-16 ***
Species	41	1.42E+09	34506456	138.705	<2e-16 ***
Life stages × Topography	6	7.37E+07	12283062	49.374	<2e-16 ***
Species × Topography	123	2.65E+08	2157010	8.67	<2e-16 ***
Life stages × Species	82	1.69E+09	20603944	82.821	<2e-16 ***
Life stages × Species × Topography	246	3.12E+08	1266914	5.093	<2e-16 ***

Df, degree of freedom.

*** represents significant effects at a level of 0.001.

**Figure 5:**
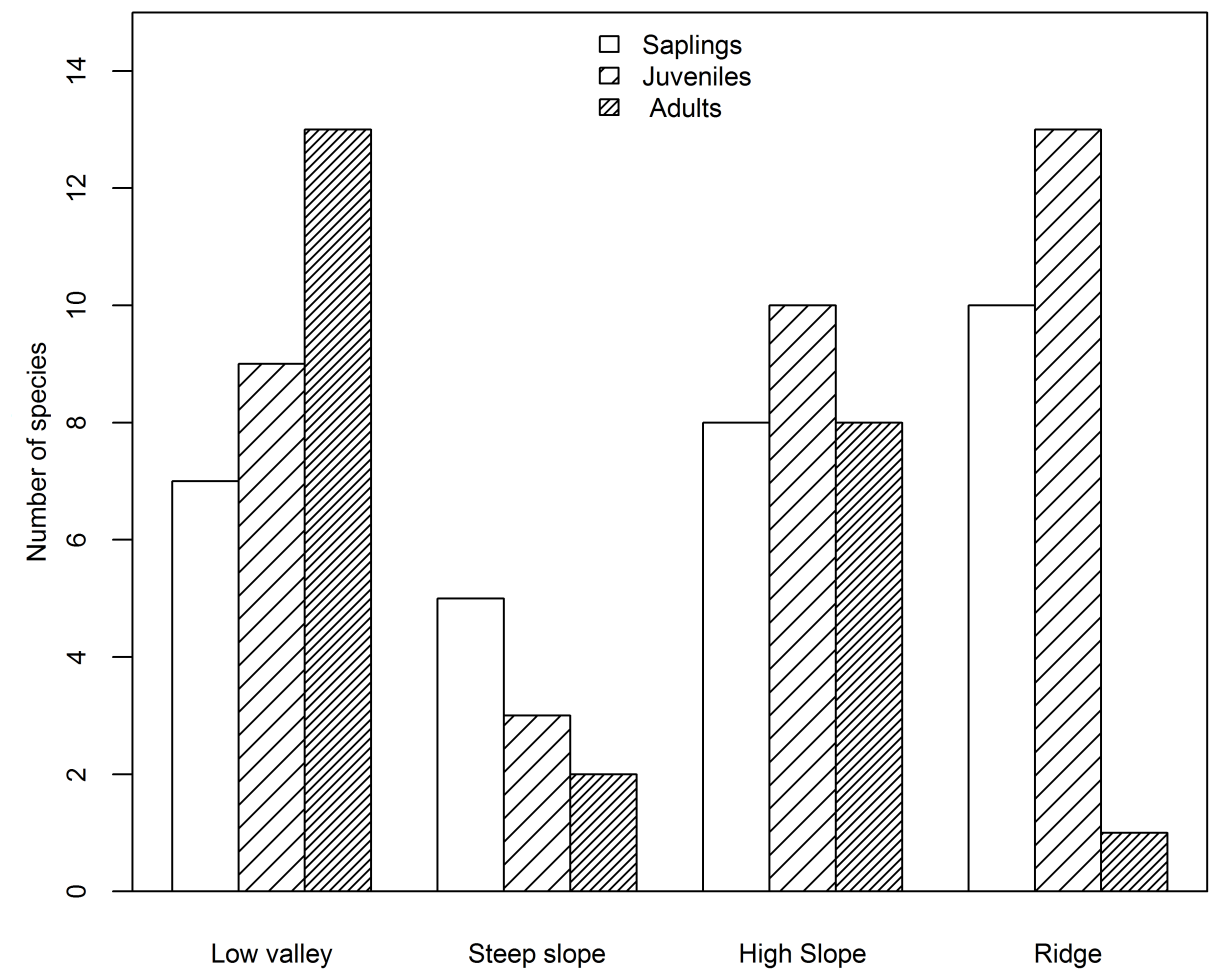
Number of species positively associated with each topography type at the sapling, juvenile and adult stages in the Jianfengling FDP.

**Figure 6:**
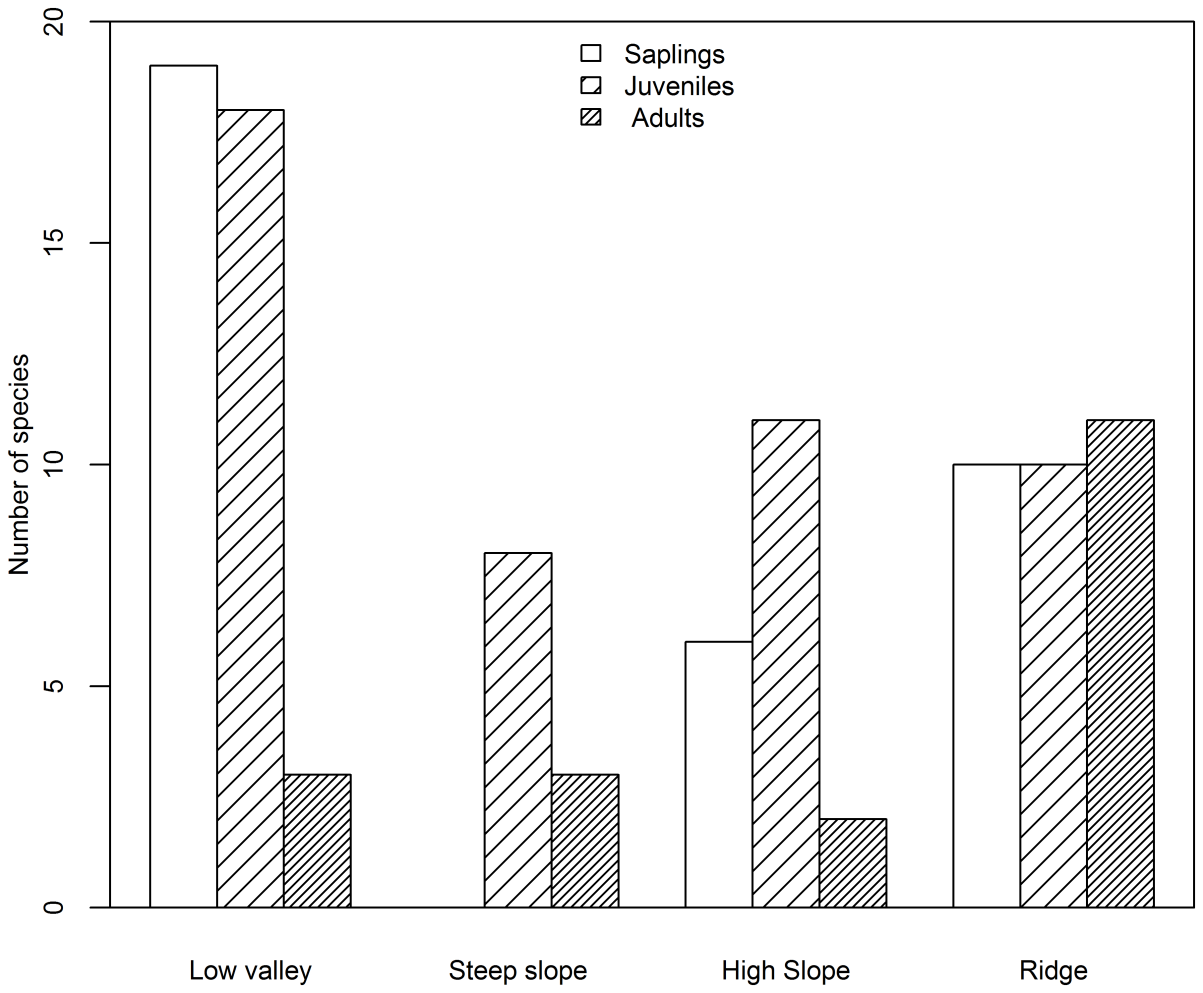
Number of species negatively associated with each topography type at the sapling, juvenile and adult stages in the Jianfengling FDP.

Based on species AGB distribution, the number of associations with each topography differed among the three life stages (**Figures 5**, **6**). At the sapling and juvenile stages, more positive associations were found in the ridge than in the other three topographies, but at the adult stage, the most positive associations were found in the low valley. For the saplings and juveniles, more negative associations were detected in the low valley than in the other three topographies, but for the adults more than one-half of all negative associations were detected in the ridge.

## Discussion

### AGB and its variation among topographies

There was an average AGB of 368.8 Mg ha^-1^ in the Jianfengling FDP. Compared with other tropical regions, the AGB of Jianfengling tropical mountain rainforest fell within the same range of biomass variation as the mature tropical lowland rainforests of Peninsular Malaysia (365~440 Mg ha^-1^) (Hoshizaki et al., 2004). The Jianfengling biomass was significantly greater than that of the French Guiana neo-tropical rainforest (309 Mg ha^-1^), the Panamanian tropical rainforest (281 Mg ha^-1^) (Chave et al., 2001, 2003), and also than the average biomass of the Brazilian Amazonian neo-tropical rainforest (356 Mg ha-1), the Cambodian tropical deciduous forest (189 Mg ha^-1^), and the semi-deciduous forest (244 Mg ha^-1^) and the limestone seasonal rainforest in Xishuangbanna, China (Laurence et al., 1999; Top et al., 2004; Qi and Tang, 2008). These results emphasize that Jianfengling’s primary tropical montane rainforest has high carbon storage capacity in its aboveground biomass.

In this study, AGB varied noticeably among the four topographies. Steep slope and ridge supported more AGB, while the two valleys supported less. High variation in AGB at the local scale has been demonstrated due to the effects of topography (De Castilho et al., 2006; Sattler et al., 2014; Riihimäki et al., 2017). Researchers have shown that topography can influence AGB distribution in multiple ways. Topography may affect AGB distribution by influencing local conditions, such as soil, hydrology, and light conditions (Mascaro et al., 2011; Liu et al., 2014), but also by influencing disturbance intensity (Lin et al., 2012). In this plot, the two valleys had many gullies due to high rainfall, thus there was less space available for water-intolerant plant growth (Punchi-Manage et al., 2013). In addition, light availability was lower in the valleys than in the steep slope and ridge topographies (Körner, 2007; Huang et al., 2014, 2016). This may explain why the two valleys supported less AGB and why many light-demanding species were more abundant in the steep slope and ridge topographies. For example, *Madhuca hainanensis* contributed significantly more biomass to the total AGB of steep slope and ridge than to that of the two valleys (Kruskal-Wallis test, *P*<0.01). This suggests that available light may be one key environmental factor that affects species composition and plant AGB distribution in the Jianfengling plot.

### Topographic preferences of species

Our results clearly showed that different species differed in their topographic preferences, as demonstrated by their AGB contributions. This was consistent with previous species-topography studies in other forests based on species abundance (Lin et al., 2012; Smith et al., 2016; Zuleta et al., 2020). Species differences in ecological strategies and different resource availability throughout the topographies may contribute to this pattern of biomass distribution (Yamada et al., 2007; Wang et al., 2018). Of the 42 species, 88.1% were positively or negatively associated with one or more of the different topographies. Compared with other forest dynamic plots that have been examined using similar methods, the Jianfengling FDP has a higher percentage of species demonstrating significant topography associations (Harms et al., 2001; Gunatilleke et al., 2006; Wang et al., 2011; Kanagaral et al., 2011) and this can be attributed to two main reasons: its topographic complexities and its forest structure (Lai, 2008). On the one hand, topographic complexities enhanced the environmental filtering effect, which led to a higher percentage. On the other hand, in contrast to a secondary forest, after a long period of environmental adaptation, the topographic niche differentiation of species in the Jianfengling primary forest is more obvious (Letcher et al., 2012; Jiao et al., 2012).

The niche complementarity hypothesis suggests that a greater number of species take greater advantage of the niche opportunities that are available in an environment, thus resulting in better use of available resources (Cardinale, 2011). In this plot, we found that different species’ topographic preferences varied based on biomass contribution, which indicates that species may complement each other in space, thus allowing for high diversity and high biomass/carbon storage at the whole plot scale. This may be a form of niche complementarity (Lin et al., 2012), which contributes to a high aboveground in Jianfengling FDP. Combined with ANOVA analysis results, our study suggests that choosing different species combinations for different topographies would overall help maximize biomass carbon storage in tropical forests.

### Variation in effects of topography among life stages

33 species (34 out of 38 positively associated and 31 out of 39 negatively associated species) showed inconsistent associations with a specific topography across the three life stages. This revealed that for most species, topographic preference and limitation were not consistent across multiple life stages. This also has been demonstrated by many previous studies based on species abundance (Comita et al., 2007; Lai et al., 2009; Punchi‐Manage et al., 2013). More than half of the significant positive associations between saplings and a particular topography persisted into the juvenile stage, but few of these associations were sustained into the adult stage. These results indicate that species’ ecological habitat preferences differ by developmental stage. Similarly, most of the negative associations between saplings and a particular topography were maintained in the juvenile stage, but just 23% of these sapling associations were further maintained into the adult stage. This indicates that, for most species, topographic limitation shows consistency from the sapling to the juvenile stage, but it tends to differ from the adult stage.

Some species were positively or negatively associated with the same topography across all life stages at this site. For example, the heliophilous species *Madhuca hainanensis* associated positively with the ridge and negatively with the low valley and the high valley across all three life stages. Hydrophilic species *Beilschmiedia laevis*, *Machilus cicatricose*, and *Polyosma cambodiana* associated positively with either the low valley or the high valley across all three stages. These above species are especially light-demanding or water-demanding, and consequently, the saplings, juveniles and adults remained spatially associated. Alternatively, this pattern may be attributed to seed dispersal limitation, in which saplings primarily establish near to the parent trees and thus form the same topographic associations (Webb and Peart, 2000; Lai et al., 2009). The role that these species, consistent in their topographic preferences across life stages, play in community assembly and forest carbon management requires further study.

In terms of the number of associations with each topography, more negative associations were detected at the sapling and juvenile stages in the low valley than in the other three topographies. This was probably due to lower light availability in the low valley, resulting from the shelter of the surrounding mountains and the greater density of large trees (DBH≥50 cm). On the contrary, more positive associations were found at the adult stage in the low valley than in the other three topographies, which may have resulted from the higher soil water content and the higher soil nitrogen, phosphorus and potassium (Xu et al., 2015b; Wang et al., 2018). In addition, the study site was in an area that is frequently hit by typhoons. In this case, direct damage mainly influences the tree layer, and large-diameter individuals are the most severely damaged (Xu et al., 2008). Because the ridge is often more susceptible to typhoons, the notably higher number of negative associations and the notably lower number of positive associations of species with the ridge were observed in the adult stage.

Furthermore, given the associations between species and topography across different life stages, our analysis showed that in the low valley, more adults than other life stages of *Alseodaphne hainanensis* and *Canarium album* led to a higher AGB, while more saplings and juveniles than adults of *Alseodaphne hainanensis* led to a lower AGB. In the ridge, more juveniles than other life stages of *Dacrydium pectinatum* and *Lithocarpus fenzelianus* contributed to a higher AGB, while more adults than other life stages did not relate to greater AGB. Combined with the results of ANOVA analysis, our results suggest that choosing different combinations of different species and life stages for different topographies is important for maximizing overall carbon storage when managing tropical montane rainforests.

## Conclusion

Working in a highly diverse primary montane rainforest, we found that the average aboveground biomass was 368.79 Mg ha^-1^, but it varied noticeably among the four topographies. AGB was significantly lower in the two valleys than in the two other topographies. Most of the 42 dominant species varied in their topographic preferences and further exhibited inconsistent topographic preferences across life stages. Our results strongly demonstrate the importance of topography and life stage in species biomass distribution, and we suggest that land managers consider both topography and life stage to best maximize carbon storage in forests.

## Data Availability

The raw data supporting the conclusions of this article will be made available by the authors, without undue reservation.

## References

[B1] Bezdek J. C. (2013). Pattern recognition with fuzzy objective function algorithms (New York: Springer Science and Business Media).

[B2] Cardinale B. J. (2011). Biodiversity improves water quality through niche partitioning. Nature 472, 86–89. doi: 10.1038/nature09904 21475199

[B3] Chave J. Condit R. Lao S. Caspersen J. P. Foster R. B. Hubbell S. P. (2003). Spatial and temporal variation of biomass in a tropical forest: results from a large census plot in Panama. J. Ecol. 91, 240–252. doi: 10.1046/j.1365-2745.2003.00757.x

[B4] Chave J. Riéra B. Dubois M. A. (2001). Estimation of biomass in a neotropical forest of French Guiana: spatial and temporal variability. J. Trop. Ecol. 17, 79–96. doi: 10.1017/S0266467401001055

[B5] Colgan M. S. Asner G. P. (2014). Coexistence and environmental filtering of species-specific biomass in an African savanna. Ecology 95, 1579–1590. doi: 10.1890/13-1160.1 25039222

[B6] Comita L. S. Condit R. Hubbell S. P. (2007). Developmental changes in habitat associations of tropical trees. J. Ecol. 95, 482–492. doi: 10.1111/j.1365-2745.2007.01229.x

[B7] Cui W. Zheng X. (2016). Partitioning tree species diversity and developmental changes in habitat associations in a subtropical evergreen broadleaf secondary forest in Southern China. Forests 7, 228. doi: 10.3390/f7100228

[B8] Deal R. L. Cochran B. Larocco G. (2012). Bundling of ecosystem services to increase forestland value and enhance sustainable forest management. For. Policy Econ. 17, 69–76. doi: 10.1016/j.forpol.2011.12.007

[B9] De Castilho C. V. Magnusson W. E. Rnode A. Rcc L. Luizao F. J. Lima A. P.. (2006). Variation in aboveground tree live biomass in a central Amazonian forest: effects of soil and topography. For. Ecol. Manage. 234, 85–96. doi: 10.1016/j.foreco.2006.06.024

[B10] Field C. B. Behrenfeld M. J. Randerson J. T. Falkowski P. (1998). Primary production of the biosphere: integrating terrestrial and oceanic components. Science 281, 237–240. doi: 10.1126/science.281.5374.237 9657713

[B11] Fortunel C. Lasky J. R. Uriarte M. Valencia R. Wright S. J. Garwood N. C.. (2018). Topography and neighborhood crowding can interact to shape species growth and distribution in a diverse Amazonian forest. Ecology 99, 2272–2283. doi: 10.1002/ecy.2441 29975420

[B12] Gong Q. Cheng J. Mi X. Chen S. Fang T. (2007). Habitat associations of wood species in the Gutianshan subtropical broad-leaved evergreen forest. Soil Water Conserv. China 5, 79–83. doi: 10.3969/j.issn.1672-3007.2007.03.014

[B13] Grubb P. J. (1977). The maintenance of species-richness in plant communities: the importance of the regeneration niche. Biol. Rev. 52, 107–145. doi: 10.1111/j.1469-185X.1977.tb01347.x

[B14] Gunatilleke C. V. S. Gunatilleke I. N. U. N. Esufali S. Harms K. E. Ashton P. M. S. Burslem D. F. R. P.. (2006). Species-habitat associations in a Sri Lankan dipterocarp forest. J. Trop. Ecol. 22, 371–384. doi: 10.1017/S0266467406003282

[B15] Guo Y. Wang B. Xiang W. Ding T. Lu S. Wen S.. (2015). Sprouting characteristics of tree species in 15-hm^2^ plot of northern tropical karst seasonal rain forest in Nonggang, Guangxi, southern China. Chin. J. Ecol. 60, 558–560. doi: 10.13292/j.1000-4890.20150311.067

[B16] Harms K. E. Condit R. Hubbell S. P. Foster R. B. (2001). Habitat associations of trees and shrubs in a 50-ha neotropical forest plot. J. Ecol. 89, 947–959. doi: 10.1111/j.1365-2745.2001.00615.x

[B17] Hoshizaki K. Niiyama K. Kimura K. Yamashita T. Bekku Y. Okuda T.. (2004). Temporal and spatial variation of forest biomass in relation to stand dynamics in a mature, lowland tropical rainforest, Malaysia. Ecol. Res. 19, 357–363. doi: 10.1111/j.1440-1703.2004.00645.x

[B18] Huang F. Ding T. Li X. Guo Y. Wang B. Xiang W. (2016). Species diversity for various associations along an altitudinal gradient in the karst seasonal rainforest in Nonggang. For. Ecol. Manage. 36, 4509–4517. doi: 10.5846/stxb201411232324

[B19] Huang F. Wang B. Ding T. Xiang W. Li X. Zhou A. (2014). Numerical classification of associations in a northern tropical karst seasonal rain forest and the relationships of these associations with environmental factors. Biodiversity Sci. 27, 295–298. doi: 10.3724/SP.J.1003.2014.10155

[B20] Jiao J. Zhang Z. Bai W. Jia Y. Wang N. (2012). Assessing the ecological success of restoration by afforestation on the Chinese Loess Plateau. Restor. Ecol. 20, 240–249. doi: 10.1111/j.1526-100X.2010.00756.x

[B21] Jin Y. Russo S. E. Yu M. (2018). Effects of light and topography on regeneration and coexistence of evergreen and deciduous tree species in a Chinese subtropical forest. J. Ecol. 106, 1634–1645. doi: 10.1111/1365-2745.12911

[B22] Jucker T. Bongalov B. Burslem D. Nilus R. Dalponte M. Lewis S. L.. (2018). Topography shapes the structure, composition and function of tropical forest landscapes. Ecol. Lett. 21, 989–1000. doi: 10.1111/ele.12964 29659115 PMC6849614

[B23] Kanagaral R. Wiegand T. Comita L. S. Huth A. (2011). Tropical tree species assemblages in topographical habitats change in time and with life stage. J. Ecol. 99, 1441–1452. doi: 10.1111/j.1365-2745.2011.01878.x

[B24] King D. A. Davies S. J. Noor N. S. (2006). Growth and mortality are related to adult tree size in a Malaysian mixed dipterocarp forest. For. Ecol. Manage. 223, 152–158. doi: 10.1016/j.foreco.2005.10.066

[B25] Körner C. (2007). The use of 'altitude' in ecological research. Trends Ecol. Evol. 22, 569–574. doi: 10.1016/j.tree.2007.09.006 17988759

[B26] Lai J. (2008). Species habitat associations and species coexistence on evergreen broadleaved forest in Gutianshan, Zhejiang. Ph.D. thesis. Chinese Academy of Sciences, Beijing.

[B27] Lai J. Mi X. Ren H. Ma K. (2009). Species-habitat associations change in a subtropical forest of China. J. Veg. Sci. 20, 415–423. doi: 10.1111/j.1654-1103.2009.01065.x

[B28] Lan G. Hu Y. Min C. Hua Z. (2011). Topography related spatial distribution of dominant tree species in a tropical seasonal rain forest in China. For. Ecol. Manage. 262, 1507–1513. doi: 10.1016/j.foreco.2011.06.052

[B29] Laurance W. F. Fearnside P. M. Laurance S. G. Delamonica P. Lovejoy T. E. Rankin-de J. M.. (1999). Relationship between soils and Amazon forest biomass: a landscape-scale study. For. Ecol. Manage. 118, 127–138. doi: 10.1016/S0378-1127(98)00494-0

[B30] Letcher S. G. Chazdon R. L. Andrade A. C. S. Bongers F. Breugel M. V. Finegan B.. (2012). Phylogenetic community structure during succession: evidence from three neotropical forest sites. Perspect. Plant Ecol. Evol. Syst. 14, 79–87. doi: 10.1016/j.ppees.2011.09.005

[B31] Li Y. Chen B. Zhou G. (2002). Research and conservation of tropical forest and the biodiversity-a special reference to Hainan island, China (Beijing: China Forestry Publishing House).

[B32] Lin D. Lai J. Muller-Landau H. C. Mi X. Ma K. (2012). Topographic variation in aboveground biomass in a subtropical evergreen broad-leaved forest in China. PloS One 7, e48244. doi: 10.1371/journal.pone.0048244 23118961 PMC3484055

[B33] Liu J. Tan Y. Slik J. W. F. (2014). Topography related habitat associations of tree species traits, composition and diversity in a Chinese tropical forest. For. Ecol. Manage. 330, 75–81. doi: 10.1016/j.foreco.2014.06.045

[B34] Lorenz K. Lal R. (2010). Carbon Sequestration in Forest Ecosystems (New York: Springer). doi: 10.1007/978-90-481-3266-9

[B35] Mascaro J. Asner G. P. Mullerlandau H. C. Breugel M. V. Hall J. Dahlin K. (2011). Controls over aboveground forest carbon density on Barro Colorado Island, Panama. Biogeosciences 8, 1615–1629. doi: 10.5194/bg-8-1615-2011

[B36] O'Brien M. J. Escudero A. (2022). Topography in tropical forests enhances growth and survival differences within and among species via water availability and biotic interactions. Funct. Ecol. 36, 686–698. doi: 10.1111/1365-2435.13977

[B37] Oksanen J. (2011). Multivariate analysis of ecological communities in R: vegan tutorial. R Package version 1, 1–43.

[B38] Oktavia D. Jin G. (2019). Species-habitat association affects demographic variation across different life stages in an old-growth temperate forest. Perspect. Plant Ecol. Evol. Syst. 40, 125482. doi: 10.1016/j.ppees.2019.125482

[B39] Pan Y. Birdsey R. A. Fang J. Houghton R. Kauppi P. E. Kurz W. A.. (2011). A large and persistent carbon sink in the world’s forests. Science 333, 988–993. doi: 10.1126/science.1201609 21764754

[B40] Punchi-Manage R. Getzin S. Wiegand T. Kanagaraj R. Gunatilleke C. V. S. Gunatilleke I. A. U.. (2013). Effects of topography on structuring local species assemblages in a Sri Lankan mixed dipterocarp forest. J. Ecol. 101, 149–160. doi: 10.1111/1365-2745.12017 24669731

[B41] Qi J. Tang J. (2008). Biomass and its allocation pattern of monsoon rain forest over limestone in Xishuangbanna of Southwest China. Chin. J. Ecol. 27, 167–177. Available online at: http://ir.xtbg.org.cn/handle/353005/2924.

[B42] Queenborough S. A. Burslem D. F. R. P. Garwood N. C. Valencia R. (2007). Habitat niche partitioning by 16 species of Myristicaceae in Amazonian Ecuador. Plant Ecol. 192, 193–207. doi: 10.1007/s11258-007-9328-3

[B43] Ramachandran A. Huxley J. D. McFaul S. Schauer L. Diez J. Boone R.. (2023). Integrating ontogeny and ontogenetic dependency into community assembly. J. Ecol. 111, 1561–1574. doi: 10.1111/1365-2745.14132

[B44] Ratcliffe S. Wirth C. Jucker T. van der Plas F. Scherer-Lorenzen M. Verheyen K.. (2017). Biodiversity and ecosystem functioning relations in European forests depend on environmental context. Ecol. Lett. 20, 1414–1426. doi: 10.1111/ele.12849 28925074

[B45] Riihimäki H. Heiskanen J. Luoto M. (2017). The effect of topography on arctic-alpine aboveground biomass and NDVI patterns. Int. J. Appl. Earth Obs. Geoinf. 56, 44–53. doi: 10.1016/j.jag.2016.11.005

[B46] Sattler D. Murray L. T. Kirchner A. Lindner A. (2014). Influence of soil and topography on aboveground biomass accumulation and carbon stocks of afforested pastures in South East Brazil. Ecol. Eng. 73, 126–131. doi: 10.1016/j.ecoleng.2014.09.003

[B47] Silvertown J. (2004). Plant coexistence and the niche. Trends Ecol. Evol. 19, 605–611. doi: 10.1016/j.tree.2004.09.003

[B48] Smith L. A. Eissenstat D. ,. M. Kaye M. W. (2016). Variability in aboveground carbon driven by slope aspect and curvature in an eastern deciduous forest, USA. Can. J. For. Res. 47, 149–158. doi: 10.1139/cjfr-2016-0147

[B49] Top N. Mizoue N. Kai S. (2004). Estimating forest biomass increment based on permanent sample plots in relation to woodfuel consumption: a case study in Kampong Thom Province, Cambodia. J. For. Res. 9, 117–123. doi: 10.1007/s10310-003-0064-9

[B50] van der Plas F. (2019). Biodiversity and ecosystem functioning in naturally assembled communities. Biol. Rev. 94, 1220–1245. doi: 10.1111/brv.12499 30724447

[B51] Wang J. (2017). Effects of topographic heterogeneity on quantity characteristic of woody plants community in Jianfengling tropical montane rainforest. M.S. thesis. Chinese Academy of Forestry, Beijing

[B52] Wang J. Liu H. Yang Q. Shen G. Zhu X. Xu Y.. (2023). Topography and structural diversity regulate ecosystem multifunctionality in a subtropical evergreen broadleaved forest. Front. For. Glob. Change 6. doi: 10.3389/ffgc.2023.1309660

[B53] Wang W. Luo Z. Zhou R. Xu D. Ai J. Ding B. (2011). Habitat associations of woody plant species in Baishanzu subtropical broad-leaved evergreen forest. Biodiversity Sci. 19, 134–142. doi: 10.3724/SP.J.1003.2011.09295

[B54] Wang J. Xu H. Li Y. Lin M. Zhou Z. Luo T.. (2018). Effects of topographic heterogeneity on community structure and diversity of woody plants in Jianfengling tropical montane rainforest. Sci. Silvae Sin. 54, 1–11. doi: 10.11707/j.1001-7488.20180101

[B55] Webb C. O. Peart D. R. (2000). Habitat associations of trees and seedlings in a Bornean rain forest. J. Ecol. 88, 464–478. doi: 10.1046/j.1365-2745.2000.00462.x

[B56] Werner F. A. Homeier J. (2015). Is tropical montane forest heterogeneity promoted by a resource-driven feedback cycle? Evidence from nutrient relations, herbivory and litter decomposition along a topographical gradient. Funct. Ecol. 29, 430–440. doi: 10.1111/1365-2435.12351

[B57] Xu H. Li Y. Lin M. Wu J. Luo T. Zhou Z.. (2015a). Community characteristics of a 60 ha dynamics plot in the tropical montane rain forest in Jianfengling, Hainan island. Biodiversity Sci. 23, 192–201. doi: 10.17520/biods.2014157

[B58] Xu H. Li Y. Luo T. Chen D. Lin M. Li Y.. (2015b). Jianfengling tropical mountain rain forest dynamic plot: community characteristics, tree species and their distribution patterns (Beijing: China Forestry Publishing House).

[B59] Xu H. Li Y. Luo T. Lin M. Chen D. Mo. J.. (2008). Influence of typhoon damrey on the tropical montane rain forest community in Jianfengling, Hainan island, China. Chin. J. Plant Ecol. 32, 1323–1334. doi: 10.3773/j.issn.1005-264x.2008.06.013

[B60] Yamada T. Zuidema P. A. Itoh A. Yamakura T. Ohkubo T. Kanzaki M.. (2007). Habitat associations of woody plant species in Baishanzu subtropical broad-leaved evergreen forest. J. Ecol. 95, 332–342. doi: 10.1111/j.1365-2745.2006.01209.x

[B61] Yao J. Bachelot B. Meng L. Qin J. Zhao X. Zhang C. (2020). Abiotic niche partitioning and negative density dependence across multiple life stages in a temperate forest in northeastern China. J. Ecol. 108, 1299–1310. doi: 10.1111/1365-2745.13335

[B62] Yuan Z. Ali A. Ruiz-Benito P. Jucker T. Mori A. S. Wang S.. (2020). Above-and below-ground biodiversity jointly regulate temperate forest multifunctionality along a local-scale environmental gradient. J. Ecol. 108, 2012–2024. doi: 10.1111/1365-2745.13378

[B63] Zeng Q., L, Y. Chen B. Wu Z. Zhou G. (1997). Research and management of tropical forest ecosystem (Beijing: China Forestry Publishing House).

[B64] Zhao P. Lu D. Wang G. Wu C. Huang Y. Yu S. (2016). Examining spectral reflectance saturation in Landsat imagery and corresponding solutions to improve forest aboveground biomass estimation. Remote Sens. 8, 469. doi: 10.3390/rs8060469

[B65] Zhou Z. Li Y. Lin M. Chen D. Xu H. Luo T. (2009). Climate changes characteristics over tropical mountain rainforest in Jianfengling during the recent 26 years: radiation, moisture, and wind factors. Acta Ecol. Sin. 29, 1112–1120. doi: 10.3969/j.issn.1673-503X.2009.03.014

[B66] Zuleta D. Muller-Landau H. C. Duque A. Caro N. Cardenas D. Castano N.. (2022). Interspecific and intraspecific variation of tree branch, leaf and stomatal traits in relation to topography in an aseasonal Amazon forest. Funct. Ecol. 36, 2955–2968. doi: 10.1111/1365-2435.14199

[B67] Zuleta D. Russo S. E. Barona A. Barreto-Silva J. S. Cardenas D. Castaño N.. (2020). Importance of topography for tree species habitat distributions in a terra firme forest in the Colombian Amazon. Plant Soil 450, 133–149. doi: 10.1007/s11104-018-3878-0

