# Supplementary material for: Topography and life stage regulate species aboveground biomass distribution in combination in a tropical montane rainforest
Source: Front Plant Sci. 2024 Sep 2;15:1403048. doi: 10.3389/fpls.2024.1403048 (PMC11402657; doi:10.3389/fpls.2024.1403048)
Supplement: Supplementary file 1 [file Table1.docx]

Table S1 Relative contribution to total aboveground biomass (AGB) per topography for 42 dominant species.

| Species Code | Jianfengling FDP | Low valley | Steep slope | Upper valley | Ridge |
| --- | --- | --- | --- | --- | --- |
| 1 | 9.20 | 7.36 | 10.47 | 10.72 | 7.83 |
| 2 | 6.94 | 3.59 | 7.50 | 5.70 | 9.49 |
| 3 | 4.43 | 4.99 | 4.44 | 4.58 | 3.94 |
| 4 | 4.42 | 4.41 | 4.90 | 4.69 | 3.73 |
| 5 | 4.00 | 3.01 | 3.97 | 4.69 | 4.12 |
| 6 | 3.58 | 4.58 | 3.09 | 3.91 | 3.17 |
| 7 | 3.28 | 5.83 | 3.60 | 1.65 | 2.62 |
| 8 | 2.71 | 1.57 | 3.27 | 2.03 | 3.40 |
| 9 | 2.67 | 2.65 | 2.61 | 3.64 | 1.98 |
| 10 | 2.56 | 0.48 | 2.25 | 0.93 | 5.52 |
| 11 | 2.45 | 1.76 | 2.90 | 0.86 | 3.71 |
| 12 | 2.21 | 1.79 | 2.16 | 2.16 | 2.58 |
| 13 | 2.11 | 2.37 | 1.89 | 2.41 | 1.94 |
| 14 | 2.00 | 2.71 | 1.68 | 2.84 | 1.21 |
| 15 | 1.78 | 1.88 | 1.98 | 1.44 | 1.78 |
| 16 | 1.74 | 0.69 | 2.19 | 1.28 | 2.33 |
| 17 | 1.73 | 1.73 | 2.21 | 1.29 | 1.59 |
| 18 | 1.62 | 0.91 | 2.34 | 0.89 | 1.91 |
| 19 | 1.52 | 2.11 | 1.64 | 1.23 | 1.28 |
| 20 | 1.43 | 0.55 | 1.81 | 0.75 | 2.15 |
| 21 | 1.36 | 1.61 | 0.77 | 2.67 | 0.77 |
| 22 | 1.23 | 0.67 | 1.24 | 1.20 | 1.60 |
| 23 | 1.13 | 1.07 | 0.90 | 1.25 | 1.31 |
| 24 | 1.00 | 0.52 | 1.47 | 0.69 | 1.07 |
| 25 | 0.97 | 0.60 | 0.85 | 1.09 | 1.22 |
| 26 | 0.95 | 1.69 | 0.69 | 1.40 | 0.39 |
| 27 | 0.89 | 1.23 | 0.73 | 0.91 | 0.84 |
| 28 | 0.81 | 1.04 | 1.02 | 0.85 | 0.42 |
| 29 | 0.81 | 2.58 | 0.26 | 0.72 | 0.33 |
| 30 | 0.78 | 1.25 | 0.37 | 1.32 | 0.46 |
| 31 | 0.77 | 0.35 | 0.67 | 0.89 | 1.05 |
| 32 | 0.74 | 0.48 | 0.84 | 0.79 | 0.77 |
| 33 | 0.74 | 0.59 | 0.68 | 0.45 | 1.11 |
| 34 | 0.72 | 0.34 | 0.59 | 0.89 | 0.96 |
| 35 | 0.72 | 0.93 | 0.50 | 1.21 | 0.40 |
| 36 | 0.70 | 0.62 | 0.47 | 1.36 | 0.46 |
| 37 | 0.68 | 1.62 | 0.73 | 0.48 | 0.18 |
| 38 | 0.67 | 0.72 | 0.36 | 1.02 | 0.66 |
| 39 | 0.66 | 1.02 | 0.58 | 0.84 | 0.37 |
| 40 | 0.62 | 1.52 | 0.46 | 0.68 | 0.18 |
| 41 | 0.55 | 1.07 | 0.13 | 1.06 | 0.26 |
| 42 | 0.41 | 0.96 | 0.15 | 0.61 | 0.16 |

Species codes are defined in Table 3.

Table S2 Species-topography associations using torus-translation tests

| Species Code | Low valley | Steep slope | Upper valley | Ridge |
| --- | --- | --- | --- | --- |
| 1 |  |  |  |  |
| 2 | -- |  |  | ++ |
| 3 | ++ |  |  | -- |
| 4 |  | + |  | -- |
| 5 | - |  |  |  |
| 6 |  |  |  |  |
| 7 |  |  |  |  |
| 8 | -- | + | - | + |
| 9 |  |  | ++ | - |
| 10 | -- |  | -- | ++ |
| 11 |  |  | -- | ++ |
| 12 | - |  |  | + |
| 13 |  | - | + |  |
| 14 | ++ |  | ++ | -- |
| 15 |  |  | - |  |
| 16 | -- |  |  | + |
| 17 |  |  |  |  |
| 18 | - | + | -- |  |
| 19 | ++ |  |  |  |
| 20 | - |  | - | + |
| 21 |  |  | ++ |  |
| 22 | -- |  |  | ++ |
| 23 |  |  |  |  |
| 24 | - | + |  |  |
| 25 | - |  |  | + |
| 26 | ++ |  | + | -- |
| 27 | + |  |  |  |
| 28 |  |  |  | -- |
| 29 | ++ | -- |  | - |
| 30 | + | -- | + | - |
| 31 | -- |  |  |  |
| 32 | - |  |  |  |
| 33 |  |  | -- | ++ |
| 34 | -- |  |  |  |
| 35 |  | - | ++ | -- |
| 36 |  |  | ++ | - |
| 37 | ++ |  |  | -- |
| 38 |  | - |  |  |
| 39 | ++ |  | ++ | -- |
| 40 | ++ |  |  | -- |
| 41 |  | -- | + |  |
| 42 | ++ | - |  | - |

Species codes are defined in Table 3. ‘+’ and ‘++’ respectively indicate significant positive associations at levels of 0.05 and 0.01, and ‘-’ and ‘--’ respectively indicate significant negative associations at levels of 0.05 and 0.01.
